# Only bioactive forms of PTH (n-oxPTH and Met18(ox)-PTH) inhibit synthesis of sclerostin – evidence from in vitro and human studies

**DOI:** 10.1007/s00424-024-02928-x

**Published:** 2024-02-23

**Authors:** Mei Li, Ahmed A. Hasan, Chang Chu, Johann-Georg Hocher, Yvonne Liu, Xiaoli Zhang, Xin Chen, Benito Yard, Bernhard K. Krämer, Berthold Hocher

**Affiliations:** 1 Fifth Department of Medicine (Nephrology/Endocrinology/Rheumatology/Pneumology), University Medical Centre Mannheim, University of Heidelberg, Heidelberg, Germany; 2 Institute of Pharmacy, Freie Universität Berlin, Berlin, Germany; 3 Charité - Universitätsmedizin Berlin, Berlin, Germany; 4 Reproductive, Genetic Hospital of CITIC-Xiangya, Changsha, China; 5 Institute of Medical Diagnostics, IMD Berlin-Potsdam, Berlin, Germany; 6 Institute of Reproductive and Stem Cell Engineering, NHC Key Laboratory of Human Stem Cell and Reproductive Engineering, School of Basic Medical Science, Central South University, Changsha, Hunan China

**Keywords:** SOST, KTRs, Non-oxidized, Oxidized, PTH

## Abstract

Sclerostin (SOST) is produced by osteocytes and is known as a negative regulator of bone homeostasis. Parathyroid hormone (PTH) regulates calcium, phosphate as well as vitamin D metabolism, and is a strong inhibitor of SOST synthesis in vitro and in vivo. PTH has two methionine amino acids (positions 8 and 18) which can be oxidized. PTH oxidized at Met18 (Met18(ox)-PTH) continues to be bioactive, whereas PTH oxidized at Met8 (Met8(ox)-PTH) or PTH oxidized at Met8 and Met18 (Met8, Met18(di-ox)-PTH) has minor bioactivity. How non-oxidized PTH (n-oxPTH) and oxidized forms of PTH act on sclerostin synthesis is unknown. The effects of n-oxPTH and oxidized forms of PTH on SOST gene expression were evaluated in UMR106 osteoblast-like cells. Moreover, we analyzed the relationship of SOST with n-oxPTH and all forms of oxPTH in 516 stable kidney transplant recipients using an assay system that can distinguish in clinical samples between n-oxPTH and the sum of all oxidized PTH forms (Met8(ox)-PTH, Met18(ox)-PTH, and Met8, Met18(di-ox)-PTH). We found that both n-oxPTH and Met18(ox)-PTH at doses of 1, 3, 20, and 30 nmol/L significantly inhibit SOST gene expression in vitro, whereas Met8(ox)-PTH and Met8, Met18(di-ox)-PTH only have a weak inhibitory effect on SOST gene expression. In the clinical cohort, multivariate linear regression showed that only n-oxPTH, but not intact PTH (iPTH) nor oxPTH, is independently associated with circulating SOST after adjusting for known confounding factors. In conclusion, only bioactive PTH forms such as n-oxPTH and Met18(ox)-PTH, inhibit SOST synthesis.

## Introduction

Parathyroid hormone (PTH) is a key endocrine regulator of calcium and phosphate homeostasis in vivo [9, 40]. PTH exerts its functions by binding to the PTH-1 receptor (PTH1R), which is primarily expressed on osteoblasts in bone and tubular cells in the kidneys [11]. Sclerostin (SOST) is a secreted glycoprotein product of the SOST gene, which is mainly produced by osteocytes [29]. It is known as a bone formation inhibitor [34]. The negative relationship between PTH and SOST has been reported in both preclinical and clinical studies. It was noted that PTH inhibits the transcription of SOST directly [4, 23, 31, 35, 41], and also impacts the expression of SOST protein in both in vivo and in vitro preclinical studies [4, 13, 31, 53]. In clinical studies, researchers found that circulating SOST is inversely associated with PTH in primary hyperparathyroidism (PHPT) [1, 47, 49] and postmenopausal women [12]. Additionally, a consistent correlation between SOST and PTH was also observed in dialysis patients [7, 21, 52, 54]. Taken together with all previous findings, in animal models and humans, we assume that the anabolic properties of PTH may be mediated at least in part by inhibiting SOST production. Although the molecular mechanisms of the negative relationship of PTH with SOST remain unknown, exploring this interaction may significantly improve our understanding of the effects of PTH on bones. In addition, the inverse relationship between circulating SOST and PTH in vivo should be taken into consideration when using SOST- and PTH-related agents in clinical practice.

As early as 1934, PTH was described as potentially loosing its bioactivity after oxidation [43]. However, for decades, n-oxPTH could not be discriminated from oxPTH in clinical samples using classic PTH assays such as second and third generation assays, until Hocher et al. introduced oxPTH columns, allowing n-oxPTH to be measured fairly easily [19]. Subsequently, the role of n-oxPTH in the clinical setting has been increasingly appreciated. Some studies showed that high n-oxPTH, rather than oxPTH concentrations are associated with adverse outcomes in chronic kidney disease (CKD) [51, 56], graft loss in kidney transplant recipients (KTRs) [48], and a high mortality risk in dialysis patients [42]. Circular dichroism (CD) studies of PTH found that oxidized methionine (Met) within PTH leads to conformational changes which might impact the interaction of PTH and PTH1R [58]. In contrast to non-oxidized PTH (n-oxPTH), oxidized PTH (ox-PTH) has a very low binding affinity to PTH1R due to secondary structure alterations, thus, it fails to stimulate the PTH1R to generate the second messenger cyclic adenosine monophosphate (cAMP) [19]. It is widely accepted that ox-PTH and n-oxPTH possess quite different biological properties [20]. Previously, Hocher et al. studied CKD cohorts with different degree [20]. They found that the ratios of oxPTH/n-oxPTH increases as the degree of CKD. The details are as follows: 3.24-fold in healthy controls (*n* = 89), 7.75-fold CKD at stage 2–4 (*n* = 620); 8.45-fold in end-stage renal disease patients on hemodialysis (*n* = 342); 8.47-fold in kidney transplant recipients (*n* = 602). This study is a big step for understanding the role of oxPTH in CKD patients. Taken together, the proportion of oxPTH increases from healthy controls to end-stage-kidney disease, thus uremia seems to influence the degree of PTH oxidation [20]. In addition, Hasan et al. recently reviewed many in vitro studies, which showed that oxPTH possesses reduced biological activity in many aspects compared to n-oxPTH, such as activating alkaline phosphatase, as well as relaxing guinea-pig tracheas, constricting blood vessels, stimulating cardiac action, and influencing fibroblast growth factor (FGF23) synthesis in vitro [18].

PTH (1–84) contains two Met amino acids at positions 8 (Met8-PTH) and 18 (Met18-PTH) that are prone to oxidation in vivo [19, 20]. It has been reported that Met8-PTH is less likely oxidized due to its location in a hydrophobic pocket [8] and a substantial change of secondary structure that is needed for the conversion to its oxidized form [58]. Contrastingly, Met18-PTH [8] is more likely to be oxidized and oxidation causes a relatively small conformation change [58]. Moreover, it is known that Met18(ox)-PTH has residual biological activity when tested separately [8, 45]. In addition, oxidation of both Met8 and 18 had the biggest conformation changes when compared with individual oxidations [58]. Accordingly, the bioactive sequence of different ox-PTH forms are as follows: Met18(ox)PTH > Met8(ox)PTH > Met8, Met18 (di-ox) PTH [58].

In a prior study we observed that only n-oxPTH, but not iPTH or oxPTH, statistically correlated with FGF23 in CKD patients [56]. Furthermore, this study found in in vitro experiments in UMR106 cells that different forms of oxPTH stimulate fibroblast growth factor (FGF23) with varying intensities of stimulation [56]. However, this study did not explore the association between SOST and n-oxPTH [56]. Considering the significant clinical importance of the relationship between PTH and SOST based on previous studies, we aimed to further investigate the effects of different forms and concentrations of PTH on the expression of SOST in both in vitro experiments in UMR106 rat osteoblast-like cells and in vivo studies in a cohort of kidney transplant recipients (KTRs).

## Materials and methods

### Cell cultures

UMR106 rat osteoblast-like cells (CRL-166; ATCC, USA) were cultured under standard culture conditions [2]. The medium consisted of Dulbecco’s Modified Eagle Medium (DMEM) with 4,5 g/L glucose (L-glutamine) (Invitrogen, USA), supplemented with 10% fetal calf serum (FCS) (Life Technologies, Germany), penicillin (100u/ml) and streptomycin (100ug/ml) (Sigma-Aldrich, Germany) at 5% CO_2_ and 37 °C. Cells were seeded in six-well plates (0.6 × 106 per cell). UMR106 cells were grown for 24 h and then removed from the previous medium before adding an equal volume of fresh culture medium into each well. The cells were treated with or without the different types of PTH 1–34 derivatives, as indicated, for an additional 24 h. Thereafter, the supernatant and cells were harvested for subsequent experiments.

In the study, we used the four types of PTH peptides to activate UMR106 cells, namely n-oxPTH, Met8(ox)-PTH, Met18(ox)-PTH, and Met8, Met18(di-ox)-PTH at 1, 3, 20, 30 nmol/L. In the control group no PTH peptide stimulation was used. All PTH peptides were purchased from JPT Peptide Technologies GmbH, as used by Zeng et al. recently [56]. Each experiment was repeated six times.

### Quantitative real-time polymerase chain reaction

We isolated total RNA from UMR106 cells using TRIzol reagent (Life Technologies, Germany). Then, 1.0 μg thereof was used for cDNA synthesis using a high-capacity cDNA reverse transcription kit (4368814, Fisher Scientific GmbH, Schwerte, Germany). Subsequently, 3.5 *uL* cDNA was added to quantitative real-time PCR (qRT-PCR). Next, we applied the TaqMan assays method to quantify relative transcript levels. The qRT-PCR reaction mix contained 0.5 *uL* cDNA, 10 *uL* TaqMan Advanced Master Mix (4444557, Fisher Scientific GmbH, Schwerte, Germany), 1 *uL* rat TaqMan assays, and 8.5 *uL* nuclease-free H_2_O per well according to the manufacturer’s instructions (50 °C for 2 min, 95 °C for 2 min, followed by 40 cycles of 95 °C for 1 s, 60 °C for 20 s). As for negative controls, nuclease-free water was used. The relevant rat TaqMan assays were actin (Actb, 4453320, Rn00667869_m1) and SOST (4453320, Rn00577971_m1), both from Fisher Scientific GmbH, Schwerte, Germany. The whole qRT-PCR procedure was performed using the ABI Step-One Plus Real-Time PCR Systems (Applied Biosystems, USA).

### Population

We collected clinical data and analyzed plasma samples of a kidney transplant patient cohort from the Transplant Clinic of Charité-Mitte, Berlin, Germany. This study was approved by the Ethics Commission of Charité University, Berlin, Germany.

The inclusion criteria included: (1) kidney transplant recipients, (2) recipients’ age ≧18 years, (3) stable and functional graft. The exclusion criteria were as follows: (1) graft loss, (2) acute infection, (3) acute rejection, (4) acute myocardial infarction, (5) malignancy, (6) pulmonary edema, (7) heart failure at baseline, (8) patients with incomplete data for variables such as SOST, n-oxPTH, ox-PTH, and iPTH. In total, 516 stable KTRs met these criteria and were included in the study. All participants were informed and signed the informed consent. In our study, clinical and laboratory parameters included the donor’s age, the recipients’ gender and age, time after transplantation, time on dialysis, cold ischemia time, HLA mismatches, estimated glomerular filtration rate (eGFR), underlying renal disease, fasting blood glucose as well as plasma SOST, osteoprotegerin (OPG), ox-PTH, n-oxPTH, iPTH, calcium, phosphate, creatinine, albumin, calcitriol (1,25(OH)_2_D), alkaline phosphatase, and total plasma cholesterol.

### Laboratory methods

At study entry, blood samples were collected and stored at -20 ℃ until the following measurements were made. Baseline laboratory measurements, such as fasting blood glucose, plasma calcium, phosphate, creatinine, albumin, 1,25(OH)_2_D, alkaline phosphatase, and total cholesterol were measured by standardized laboratory methods. The eGFR was calculated based on the CKD-EPI equation.

N-oxPTH was measured using the intact-PTH electrochemiluminescence immunoassay (ECLIA; Roche PTH, Intact [iPTH]), which used two types of monoclonal antibodies. One is a biotinylated monoclonal antibody, which can react with amino acids 26–32, and the other is a capture ruthenium-complexed monoclonal antibody targeting amino acids 55–64. The assay was performed on a Roche Modular E 170®. Therein, any oxidized forms of PTH (1–84) at positions of Met8 and/or Met18 were removed through a specific chromatography column where anti-human-oxPTH monoclonal antibodies were contained. Moreover, iPTH was analyzed directly by classical third generation iPTH sandwich assays (in short iPTH assay), which, as a limitation, cannot differentiate n-oxPTH from ox-PTH. Details were described in our previous studies [19, 42].

SOST concentration was measured using a commercial enzyme-linked immunosorbent assay (ELISA) (BI-20492, Biomedica Medizinprodukte GmbH &Co KG, Vienna, Austria), and OPG concentration was also measured using a commercial ELISA (BI-20403, Biomedica Medizinprodukte GmbH &Co KG, Vienna, Austria). Plasma SOST and OPG samples were measured according to the manufacturer’s instructions as described in the studies of Zeng et al. [56, 57]. Briefly, in the first step, 150 uL assay buffer was pipetted into wells, which were pre-coated with goat polyclonal anti-SOST/OPG antibodies. Then, 20 uL/well of standards, samples or controls were added in duplicate into the plates. Next, 50 uL/well of biotinylated anti-SOST/OPG antibodies were added into the plates. The plates were incubated at room temperature (RT) (18–24 °C) for 4 h for biotinylated anti-OPG antibody, but overnight in the dark for biotinylated anti-SOST antibody. After washing, a conjugate (200 uL/well) was pipetted into all wells and the plates were incubated for 1 h at room temperature (RT), then, washed as before. After a final washing, 200 uL/well of substrate solution were added into the plates, which were then incubated for 30 min at RT in the dark. The reaction was stopped by adding a stop solution (50 uL/well), and the absorbance was read at 450 nm with a correction wavelength of 630 nm using a microplate reader (Bio-Rad 680, USA).

### Statistics

The data on the relative gene expression of SOST in cultured UMR106 cells treated with the four types of PTH peptides was non-normally distributed, therefore, the Kruskal–Wallis test followed by Dunn’s post-hoc test was performed. In the clinical cohort, quantitative variables were given as medians (interquartile range, IQR) due to non-normal distribution, and categorical variables were presented as n (%). The Spearman correlation analysis was used to evaluate the correlations between plasma SOST and n-oxPTH, oxPTH, and iPTH, along with other clinical and blood biochemical parameters. Then, we selected clinical variables with *p* < 0.05, as well as iPTH and oxPTH, into a multivariate linear regression model (Method: stepwise regression analysis), where we further assessed the relationship between plasma SOST and PTH forms. All data were analyzed using SPSS software version 26.0 (IBM, New York, USA) and GraphPad Prism 9.0 (GraphPad Software, Inc. La Jolla. California, USA). Significance was defined at a two-sided *p*-value < 0.05.

## Results

### In vitro studies

To investigate how n-oxPTH, Met18(ox)-PTH, Met8-Met18(di-ox)-PTH, and Met8(ox)-PTH influence SOST gene expression, we conducted an in vitro experiment in UMR106 osteoblast-like cells.

**Fig. 1:**
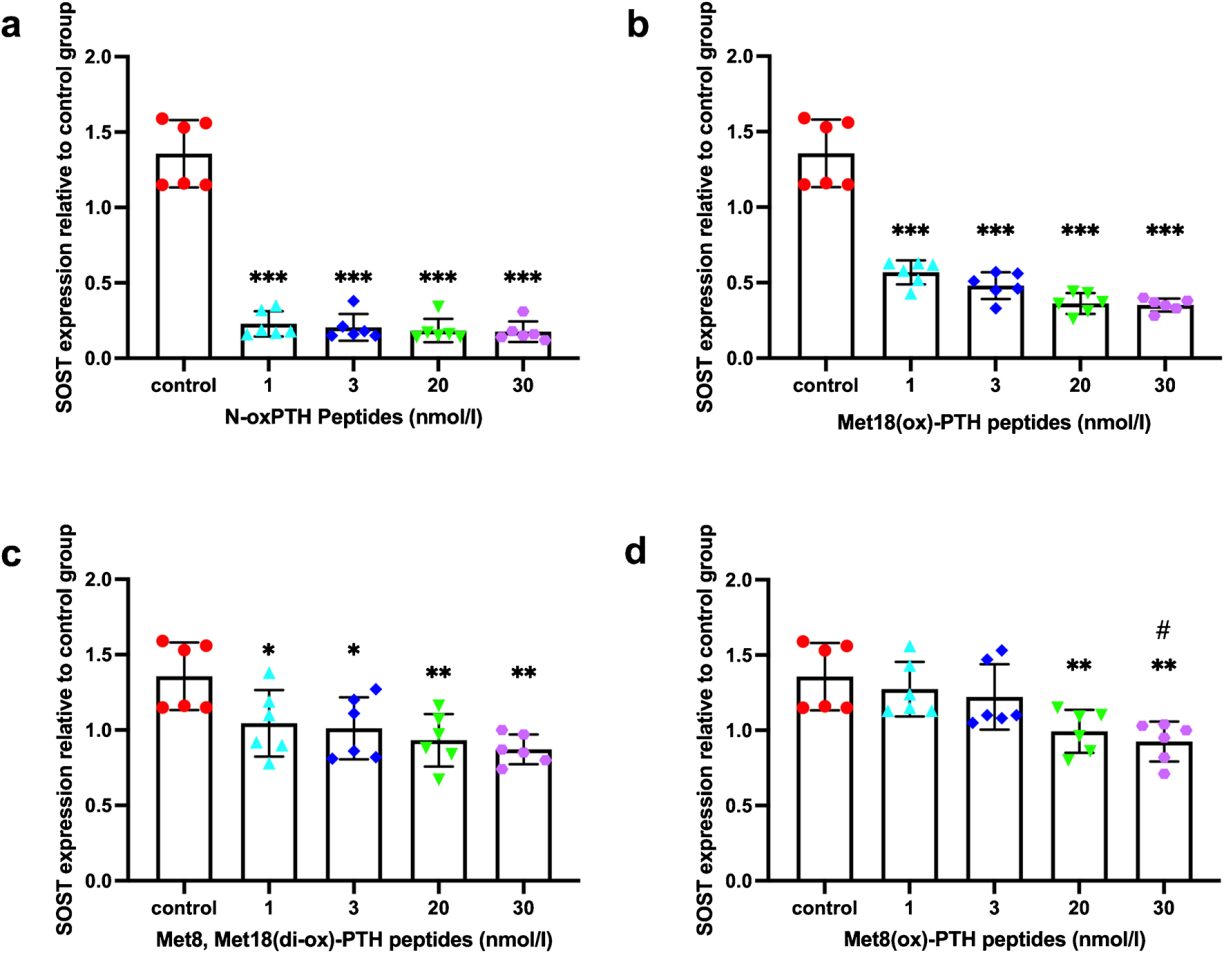
The effects of n-oxPTH and various forms of oxPTH on SOST gene expression in UMR106 rat osteoblast-like cells (**a**–**d**). We cultured the cells for the first 24 h, then, treated them with or without PTH 1–34 derivatives for another 24 h at 1, 3, 20, and 30 nmol/L, respectively. The experiment was repeated six times and the levels of SOST expression were measured by qRT-PCR. **a** and **b** Both n-oxPTH and Met18(ox)-PTH peptides, at all dosages caused a significant decrease in SOST gene expression when compared with negative controls (*P* < 0.001), however n-ox PTH had a maximum inhibitory effect already at 1 nmol/L; an extent of inhibition, that was not reached even with a dose of 30 nmol/L Met18(ox)-PTH peptides. **c** A similar trend was also observed in the Met18(di-ox)-PTH peptides, despite a relatively slight inhibition in comparison to negative controls (*P* < 0.05). **d** However, the Met8(ox)-PTH peptides inhibited the SOST mRNA synthesis even to a lesser extent and only at 20 and 30 nmol/L (*P* < 0.05), but not at 1 and 3 nmol/L, when compared with negative controls. The x-axis shows the different concentrations. The y-axis represents the relative SOST gene expression. Data was analyzed using the Kruskal–Wallis test followed by Dunn’s post-hoc test. ^*^ *P* < 0.05, ^**^ P < 0.01, ^***^ *P* < 0.001 vs. control group. ^#^ *P* < 0.05, 1 nmol/L vs. 3 nmol/L Met8(ox)-PTH peptides. Abbreviations: iPTH, intact PTH; n-oxPTH, non-oxidized PTH; oxPTH, oxidized PTH; PTH, parathyroid hormone; SOST, sclerostin

All investigated concentrations of n-oxPTH suppressed the expression of SOST significantly (*P* < 0.001). A dose-dependence was not detectable with a clear suppression of SOST expression down to a basal level already at the lowest concentration of 1 nmol/L. Met18(ox)-PTH inhibited SOST expression dose-dependently at all concentrations (1, 3, 20, 30 nmol/L) (*P* < 0.001), but the extent of this suppression was less marked than with n-oxPTH compared. Met8, Met18(di-ox)-PTH also showed a dose-dependent but comparatively much weaker inhibition of SOST expression at all concentrations. Met8(ox)-PTH, on the other hand, inhibited SOST expression even weaker and only at concentrations of 20 and 30 nmol/L. In addition, the inhibition of SOST expression was different in the distinct concentrations of Met8(ox)-PTH in the culture medium with concentrations of 1 and 30 nmol/L (*P* < 0.05). (Fig. 1 a–d).

### Clinical study

A total of 516 stable KTRs, with 314 (61%) males and 202 (39%) females, were included in this study. The median age of the cohort was 54.7 years (IQR 45.6–67.2 y). Demographics and characteristics of the study population are presented in Table 1. At study entry, the median of plasma SOST, iPTH, ox-PTH, and n-oxPTH levels were 47.0 (35.7–61.9) pmol/L, 77.3 (49.1–128.1) pg/mL, 68.7 (42.1–116.1) pg/mL, and 8.6 (5.4–12.9) pg/mL, respectively.

**Table 1:** Clinical characteristics and plasma parameters of stable renal transplant recipients

	*N* = 516
Gender	
Male	314 (61.0%)
Female	202 (39.0%)
Age at study entry (years)	54.7 (45.6–67.2)
Donor age (years)	51.0 (40.0–60.0)
Time of post-transplantation (months)	69.0 (34.7–124.3)
Time on dialysis (months)	46.0 (19.8–75.0)
Cold ischemia time (hours)	8.6 (3.3–14.1)
Plasma sclerostin (pmol/L)	47.0 (35.7–61.9)
OxPTH (pg/mL)	68.7 (42.1–116.1)
N-oxPTH (pg/mL)	8.6 (5.4–12.9)
iPTH (pg/mL)	77.3 (49.1–128.1)
eGFR (mL/min/1.73m^2^)	43.0 (32.0–58.0)
Plasma calcium (mmol/L)	2.5 (2.4–2.6)
Plasma phosphate (mmol/L)	0.8 (0.7–1.0)
OPG (pmol/L)	4.2 (3.5–5.6)
Plasma creatinine (mg/dL)	1.54 (1.25–2.0)
Plasma albumin (g/dL)	4.6 (4.4–4.8)
Plasma 1,25(OH)_2_D (pmol/L)	91.0 (59.0–131.0)
Alkaline phosphatase (U/L)	73.0 (60.0–94.0)
Total cholesterol (mg/dL)	217.0 (186.0–254.0)
Fasting blood glucose (mg/dL)	87.0 (76.0–104.0)
Underlying renal disease	
Primary kidney disease	425 (82.4%)
Secondary kidney disease	40 (7.8%)
Unknown renal disease	51(9.8%)
HLA mismatches	
MMA_broad (yes)	312 (60.5%)
MMB_broad (yes)	371 (71.9%)
MMDR broad (yes)	344 (66.7%)

Data were presented as median (interquartile range) or n (%)

Abbreviations: *eGFR* estimated glomerular filtration rate, *HLA* human leukocyte antigen, *iPTH* intact PTH, *n-oxPTH* non-oxidized PTH, *oxPTH* oxidized PTH, *PTH* parathyroid hormone, *OPG* osteoprotegerin

**Fig. 2:**
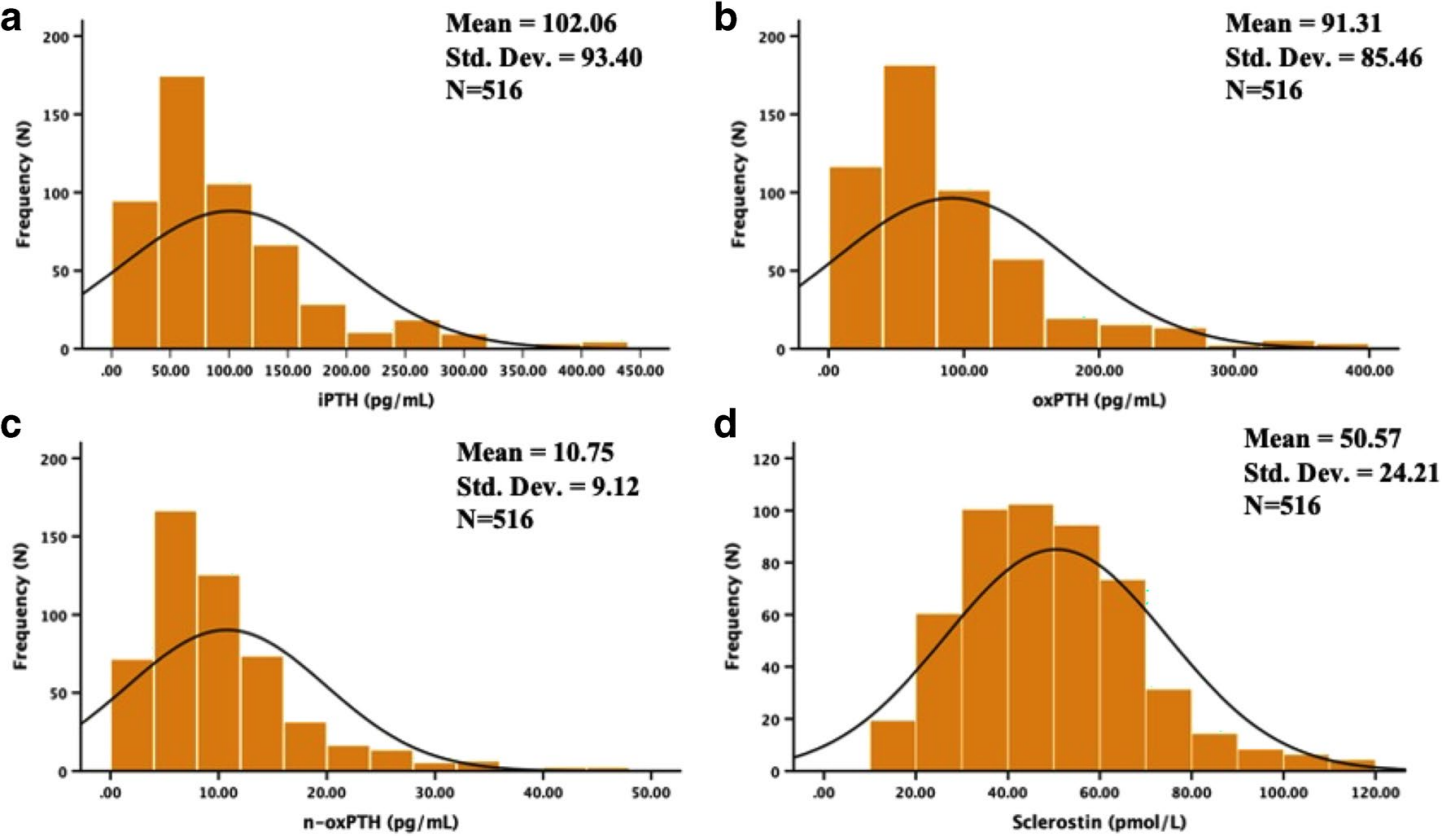
Histogram showing the frequency distribution of plasma iPTH (**a**), oxPTH (**b**), n-oxPTH (**c**), and Sclerostin (**d**) in 516 stable kidney transplant recipients. The actual concentrations of iPTH, oxPTH, and n-oxPTH in the study population are not normally distributed, except for SOST concentration, which is similar to a normal distribution. Abbreviations: iPTH, intact PTH; n-oxPTH, non-oxidized PTH; oxPTH, oxidized PTH; PTH, parathyroid hormone; SOST, sclerostin

The distribution of iPTH, oxPTH, n-oxPTH, and SOST concentrations is shown in Fig. 2. The distribution of actual iPTH, oxPTH, and n-oxPTH concentrations in the KTR cohort were not normally distributed, but the distribution of SOST concentration was nearly normally distributed. Thus, we analysed the relationship of plasma SOST to other relevant clinical and biochemical variables in adult KTRs using the Spearman correlation analysis, as described in Table 2. After primary correlation analysis, only n-oxPTH (*n* = 516, r_s_ = −0.095, *P* = 0.030), but not iPTH (*n* = 516, r_s_ = −0.066, *P* = 0.136) nor oxPTH (*n* = 516, r_s_ = −0.056, *P* = 0.202), were associated with plasma SOST (*P* < 0.05). Moreover, clinical variables including the recipients’ gender (*n* = 516, r_s_ = −0.152, *P* < 0.001), age at study entry (*n* = 515, r_s_ = 0.258, *P* < 0.001), plasma phosphate (*n* = 508, r_s_ = 0.160, *P* < 0.001), plasma creatinine (*n* = 514, r_s_ = 0.258, *P* < 0.001), eGFR (*n* = 498, r_s_ = −0.250, *P* < 0.001) and plasma OPG (*n* = 516, r_s_ = 0.235, *P* < 0.001) were correlated with plasma SOST.

**Table 2:** Spearman correlation analysis of Sclerostin with clinical and biochemical characteristics of 516 stable renal transplant recipients

	Correlation Coefficient	*P* value	*N*
Sex (male/female)	-0.152	< 0.001	516
Age at study entry (years)	0.258	< 0.001	515
Donor age (years)	0.082	0.067	501
OxPTH (pg/mL)	-0.056	0.202	516
N-oxPTH (pg/mL)	-0.095	0.030	516
iPTH (pg/mL)	-0.066	0.136	516
Phosphate (mmol/L)	0.160	< 0.001	508
Calcium (mmol/L)	-0.071	0.110	513
Albumin (g/dL)	-0.002	0.973	325
Alkaline phosphatase (U/L)	-0.030	0.502	513
Total cholesterol (mg/dL)	-0.029	0.507	511
Creatinine (mg/dL)	0.258	< 0.001	514
Time post-transplantation (months)	0.059	0.178	516
eGFR (mL/min/1.73m^2^)	-0.250	< 0.001	498
Cold ischemia time (hours)	0.079	0.078	503
OPG (pmol/L)	0.235	< 0.001	516
Fasting blood glucose (mg/dL)	0.053	0.233	512
HLA mismatches			
MMA_broad	0.063	0.069	516
MMB_broad	0.034	0.326	516
MMDR_broad	0.054	0.118	516
Time on dialysis (months)	0.008	0.872	450
Plasma 1,25(OH)_2_D (pmol/L)	-0.097	0.079	409

Abbreviations: *eGFR* estimated glomerular filtration rate, *HLA* human leukocyte antigen, *iPTH* intact PTH, *n-oxPTH* non-oxidized PTH, *oxPTH* oxidized PTH. *PTH* parathyroid hormone, *OPG* osteoprotegerin

**Fig. 3:**
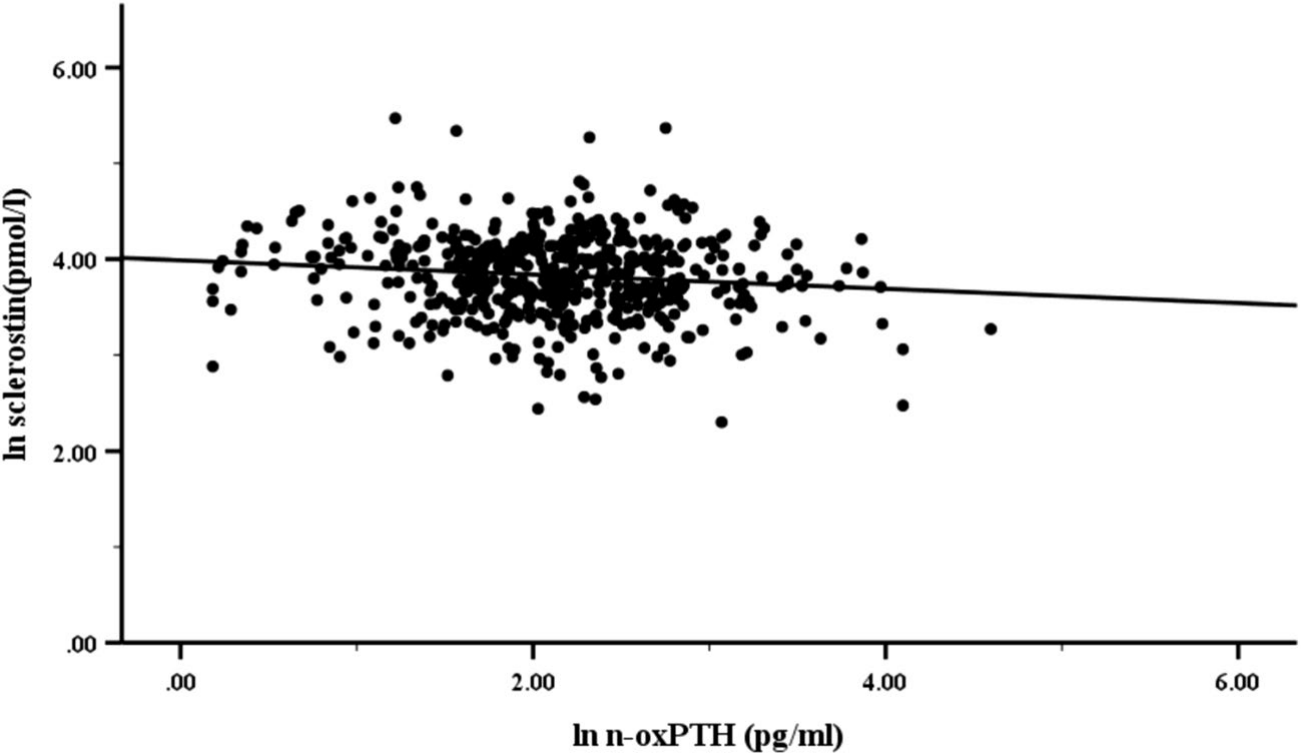
Scatterplot showing the correlation between SOST and n-oxPTH plasma concentrations in kidney transplant recipients. As actual plasma SOST and n-oxPTH concentrations were moderately skewed in the study, we calculated ln-SOST and ln-n-oxPTH. After data transformation, both ln-SOST and ln-n-oxPTH were close to being normally distributed. We used Pearson correlation analysis to assess the associations between ln-SOST and ln-n-oxPTH. Our findings showed that ln-SOST was significantly negatively associated with ln-n-oxPTH (*P* < 0.05). Abbreviations: n-oxPTH, non-oxidized PTH; PTH, parathyroid hormone; SOST, sclerostin

Due to the moderately skewed distributions of actual SOST and n-oxPTH concentrations in the study population, we calculated ln-SOST and ln-n-oxPTH to achieve a similar normal distribution (ln-SOST Kolmogorov–Smirnov Test: D = 0.040, *P* = 0.048; ln-n-oxPTH Kolmogorov–Smirnov Test: D = 0.038, *P* = 0.075, respectively). Then, we further analyzed the correlation between n-oxPTH and SOST using the Pearson correlation analysis. A significant negative correlation was observed between n-oxPTH and SOST concentrations (r = −0.121, *P* < 0.05) (Fig. 3).

To further investigate the correlation of plasma SOST with n-oxPTH, we carried out a multivariate linear regression analysis using the stepwise method (Table 3). Plasma n-oxPTH, oxPTH, iPTH, recipients’ gender and age at study entry, plasma phosphate, plasma creatinine, eGFR, plasma OPG, and plasma SOST were included in the model. Our findings showed that plasma n-oxPTH (95%CI: −0.829- −0.355; *P* < 0.001), plasma creatinine (95%CI: 6.392–13.065; *P* < 0.001), recipients’ gender (95%CI: −9.657- −0.209; *P* = 0.040) and age at study entry (95%CI: 0.228–0.508; *P* < 0.001) were independently associated with the levels of circulating SOST.

**Table 3:** Stepwise multivariate linear regression of variables associated with circulating SOST in stable kidney transplant recipients

	Standardized Beta	*T*	*P*	95% confidence interval for *B*
N-oxPTH	-0.216	-4.913	< 0.001	-0.829- -0.355
Plasma creatinine	0.259	5.729	< 0.001	6.392- 13.065
Gender	-0.089	-2.062	0.040	-9.657- -0.209
Age at study entry	0.217	5.178	< 0.001	0.228- 0.508

Stepwise linear regression analysis was used to evaluate the correlations between plasma SOST and n-oxPTH, oxPTH, and iPTH. The variables with *p*-value < 0.05 including recipients’ gender and age, serum phosphate, creatinine, OPG, and eGFR were included. Additionally, plasma oxPTH and iPTH were also included into the stepwise regression analysis model

Abbreviations: *n-oxPTH* non-oxidized PTH, *OPG* osteoprotegerin, *PTH* parathyroid hormone, *SOST* sclerostin

## Discussion

This study demonstrates that non-oxidized and oxidized forms of PTH have different biological effects on SOST expression in vitro. N-oxPTH and Met18(ox)-inhibit SOST mRNA expression in UMR106 cells, whereas Met8(ox)-PTH and Met8, Met18(di-ox)-PTH don’t do this. In adult kidney transplant recipients, only n-oxPTH, but not oxPTH (a measure of all oxidized forms of PTH) nor iPTH (a measure of all PTH forms), is independently inversely associated with SOST concentrations after adjusting for known confounding factors.

In 2005, the hypothesis that PTH directly downregulates SOST expression was initially proven through in vivo and in vitro experiments [23]. A reduction of SOST expression in the femur bone was also observed in vivo [23]. Subsequently, further independent studies obtained consistent results that the addition of PTH impeded SOST expression in vitro and in vivo [4, 35, 41]. Our study is in line with the previous studies, but demonstrate for the first time that only bioactive forms of PTH (n-oxPTH as well as Met18(ox)-PTH) but not Met8(ox)-PTH and Met8, Met18(di-ox)-PTH do suppress SOST gene expression. Our finding that oxidation of PTH matters with regard to its biological activity is supported by the following points:

1. Compared to n-oxPTH, oxPTH (the authors tested a mixture of oxidized PTH forms) had a significantly lower binding affinity to PTHR, resulting in a lower biological activity of oxPTH, which presented itself as a reduction in regulation of calcium and phosphate metabolisms in vivo [20].
2. Previous studies have proven that methionine amino acids at position 18 are more likely to be oxidized in vivo than those at position 8, see [8]. Methionine residue 8 is in a hydrophobic pocket, which leads to a more difficult oxidation [45]. Only oxidized forms of PTH at position 18 still have residual biological activity, shown when the activity of oxidized forms of PTH are separately tested [14, 45]. This is because PTH with an oxidized methionine residue at position 18 has a more similar structure to n-oxPTH, which has a full biological function [45]. This evidence supports our findings that Met18(ox)-PTH has the by far strongest effects on inhibiting SOST expression among the oxidized PTH forms.

Although the mechanisms of PTH downregulating SOST are not completely understood, possible underlying mechanisms are as follows; Firstly, PTH probably inhibits SOST expression through inducing proteasomal degradation of Runx2 [5] which is a SOST-dependent gene and can upregulate sclerostin production [39]. Secondly, PTH downregulates osteocyte-specific SOST expression by inhibiting the activity and/or expression of MEF2 in osteocytes [26, 30], which is important for the transcriptional activation of the SOST bone enhancer [10, 25, 30]. Expression of MEF2 and SOST are co-localized in osteoblasts, and the expression of three types of MEF2 genes including MEF2A, MEF2C, and MEF2D has been proven to be necessary for endogenous SOST expression in UMR-106 cells [30]. Lastly, many in vitro studies have proven that PTH-induced inhibition of SOST mRNA and SOST protein levels is direct and primarily mediated by the activation of the cAMP/PKA signaling pathway downstream of PTH1R [4, 23, 26].

In the clinical cohort study, only n-oxPTH in adult KTRs, but not iPTH nor oxPTH, is independently inversely associated with SOST after multivariate linear regression analysis. The clinical data in KTRs are in good agreement with a recent study showing that plasma SOST levels are negatively correlated with PTH concentrations after, (bioactive) PTH treatment in postmenopausal women [12, 49]. Similarly, the negative association can be seen in patients with primary hyperparathyroidism [1, 31, 47]. It might be assumed – but still needs to be proven—that the parathyroid gland in patients with hyperparathyroidism mainly secretes bioactive forms of PTH, since hypercalcemia, a biological consume of increase bioactive PTH, is often seen in this patient population. In CKD patients on dialysis, an inverse relationship between SOST and PTH was also reported. However, these studies did not investigate whether this association was independent of confounding factors [7, 21, 52, 54]. In our clinical study we likewise saw an inverse relationship between iPTH and n-oxPTH on the one hand and SOST on the other hand. However, only n-oxPTH has a significant inverse correlation with SOST after adjusting confounding factors, although the R^2^ value in the Fig. 3 is low. We considered that PTH peptides are only one of many factors influencing SOST in kidney transplant patients. Additionally, it is feasible that SOST concentrations in kidney transplant recipients might be influenced by glucocorticoids [3, 6, 15, 24, 28, 32, 38, 46, 50], but only less than 10% of patients in our study received corticosteroids in an actually as low as possible dose after renal transplant. The patients in this cohort received renal transplantation a very long time ago. The policy in our center is to withdraw steroids after 1 year of kidney transplants when patients are stable because of the severe side effects of steroids. Thus, steroids play a very limited role in our study population.

Compared with previous studies, our study separately tested oxidized and non-oxidized forms of PTH in vitro and analyzed their different associations with SOST in KTRs. Our results emphasized the importance of separately measuring n-oxPTH, as a bioactive form of PTH, in the clinical setting. In this study, circulating oxPTH concentrations are much higher than that of n-oxPTH (68.7 (42.1–116.1) pg/mL vs. 8.6 (5.4–12.9) pg/mL, respectively), suggesting that the majority of PTH is oxidized in vivo. Moreover, previous studies have documented that Met18(ox)-PTH is prone to be oxidized in the organisms compared with Met8(ox)-PTH [8]. Thus, we assume that blood Met18(ox)-PTH levels are higher than that of Met8(ox)-PTH under conditions of oxidative stress as it is seen in dialysis patients, which is consistent with the hypothesis of Ursem et al. [45]. Met18(ox)-PTH had a smaller impact on conformation change than Met8(ox)-PTH did [58], resulting in the residual biological activity of Met18(ox)-PTH [44, 45]. These findings support also our in vitro finding that Met18(ox)-PTH peptide is still able to downregulate SOST generation.

In contrast to dialysis patients, in our kidney transplant population study, we did not saw a significant association of SOST with either ox-PTH or iPTH, suggesting that in this population Met8(ox)-PTH and Met8, Met18(di-ox)-PTH are the predominant forms of oxPTH in vivo and weaken this relationship. Met18(ox)-PTH with preserved bioactivity seems to play a minor role. This hypothesis, however, can only be proven with clinical assay systems that can differentiate the various oxidized forms of PTH. The clinical assay system that we used in our study is not able to do this, it just measures the sum of all oxidized forms of PTH.

It has been reported that SOST is a risk factor for abdominal aortic calcification [17] and coronary artery calcification [27]. In patients on dialysis, many studies found a relationship between SOST with cardiovascular mortality [16, 22] and all-cause mortality [16]. A possible explanation is that SOST is an inhibitor of the Wnt signaling pathway, which is related to vascular calcification and the development of cardiovascular events [37]. In addition, our prior study confirmed that SOST is independently associated with all-cause mortality in transplanted patients [57]. Over half of the KTRs died from cardiovascular event [33, 55]. These study findings indicate that controlling SOST levels in a proper range is essential in CKD patients and KTRs. Some anti-sclerostin antibodies, such as romosozumab and blosozumab, are being tested in clinical trials for the treatment of postmenopausal osteoporosis via neutralizing SOST, and the relevant trial outcomes are promising [36]. We hypothesize that a bioactive PTH-associated agent may be a promising approach to regulating SOST in kidney-related diseases to reduce the risk of cardiovascular diseases or relevant mortality. The interaction between SOST and PTH in vivo, especially n-oxPTH, should be taken into consideration when using either PTH or SOST-relevant drugs in CKD populations.

Several limitations should be considered in this study when interpreting its findings. To begin with, we could not separate various forms of oxPTH, such as Met18(ox)-PTH, Met8(ox)-PTH, and Met8, Met18(di-ox)-PTH, in the clinical study due to the restrictions of the current measuring technique. Consistent with previous findings [44, 45], our experimental data also confirmed that Met18(ox)-PTH had the biological activity in vitro. However, current techniques cannot separately measure circulating Met18(ox)-PTH, Met8(ox)-PTH, and Met8, Met18(di-ox)-PTH levels in KTRs, therefore, whether the bioactive Met18(ox)-PTH is also associated with SOST or not in vivo continues to be unclear. Secondly, we observed various effects of n-oxPTH, Met18(ox)-PTH, Met8(ox)-PTH, and Met8, Met18(di-ox)-PTH peptides on SOST mRNA expression in our in vitro experiment. However, we did not assess the effects of different PTH peptides on the SOST protein expression levels. Thus, a further study examining the effects of various forms of PTH on SOST protein levels would be recommended. Lastly, this study is from a single center and the association between n-oxPTH and SOST concentrations was analyzed only in a KTR cohort. Therefore, the findings of this study need to be verified in multiple centers and other kidney diseases in the future.

To conclude, bioactive PTH, such as n-oxPTH and Met18(ox)-PTH, markedly inhibits SOST gene expression in vitro. In the cohort study, we demonstrate an independent association between circulating n-oxPTH, but not iPTH and oxPTH, and SOST, which is consistent with our in vitro results. Given the fact that the majority of PTH in vivo is oxidized, which lacks partial or entire bioactivity, such as the corresponding Met18(ox)-PTH or Met18(ox)-PTH, thus, assay systems that can measure all bioactive forms of PTH separately would be beneficial for clinical use to make proper clinical decisions.

## Data Availability

The data that support the findings of this study are available on request from the corresponding author (B.H.).

## Abbreviations

cAMP: Cyclic adenosine monophosphate
CKD: Chronic kidney disease
DMEM: Dulbecco’s modified eagle medium
eGFR: Estimated glomerular filtration rate
HLA: Human leukocyte antigen
hPTH: Human PTH
iPTH: Intact PTH
KTR(s): Kidney transplant recipient(s)
Met: Methionine
Met8(ox)-PTH: PTH oxidized at Met8
Met18(ox)-PTH: PTH oxidized at Met18;
Met8, Met18(di-ox)-PTH: PTH oxidized at Met8 and Met18
MEF2: Myocyte enhancer factor 2
n-oxPTH: Non-oxidized PTH
ox-PTH: Oxidized PTH
OPG: Osteoprotegerin
PTH: Parathyroid hormone
SOST: Sclerostin
